# Intervention effects on physical activity: the HEIA study - a cluster randomized controlled trial

**DOI:** 10.1186/1479-5868-10-17

**Published:** 2013-02-05

**Authors:** May Grydeland, Ingunn Holden Bergh, Mona Bjelland, Nanna Lien, Lene Frost Andersen, Yngvar Ommundsen, Knut-Inge Klepp, Sigmund Alfred Anderssen

**Affiliations:** 1 Department of Nutrition, Faculty of Medicine, University of Oslo, Oslo, Norway; 2 Department of Sports Medicine, Norwegian School of Sports Sciences, PB 4014 Ullevaal Stadion, Oslo NO-0806, Norway; 3 Department of Coaching and Psychology, Norwegian School of Sport Sciences, Oslo, Norway

**Keywords:** Obesity prevention, Overweight, Accelerometers, Intervention, Children, Adolescents

## Abstract

**Background:**

Although school-based interventions to promote physical activity in adolescents have been suggested in several recent reviews, questions have been raised regarding the effects of the strategies and the methodology applied and for whom the interventions are effective. The aim of the present study was to investigate effects of a school-based intervention program: the HEalth in Adolescents (HEIA) study, on change in physical activity, and furthermore, to explore whether potential effects varied by gender, weight status, initial physical activity level and parental education level.

**Methods:**

This was a cluster randomized controlled 20 month intervention study which included 700 11-year-olds. Main outcome-variable was mean count per minute (cpm) derived from ActiGraph accelerometers (Model 7164/GT1M). Weight and height were measured objectively. Adolescents reported their pubertal status in a questionnaire and parents reported their education level on the consent form. Linear mixed models were used to test intervention effects and to account for the clustering effect of sampling by school.

**Results:**

The present study showed an intervention effect on overall physical activity at the level of p = 0.05 with a net effect of 50 cpm increase from baseline to post intervention in favour of the intervention group (95% CI −0.4, 100). Subgroup analyses showed that the effect appeared to be more profound among girls (Est 65 cpm, CI 5, 124, p = 0.03) and among participants in the low-activity group (Est 92 cpm, CI 41, 142, p < 0.001), as compared to boys and participants in the high-activity group, respectively. Furthermore, the intervention affected physical activity among the normal weight group more positively than among the overweight, and participants with parents having 13–16 years of education more positively than participants with parents having either a lower or higher number of years of education. The intervention seemed to succeed in reducing time spent sedentary among girls but not among boys.

**Conclusions:**

A comprehensive but feasible, multi-component school-based intervention can affect physical activity patterns in adolescents by increasing overall physical activity. This intervention effect seemed to be more profound in girls than boys, low-active adolescents compared to high-active adolescents, participants with normal weight compared to the overweight, and for participants with parents of middle education level as opposed to those with high and low education levels, respectively. An implementation of the HEIA intervention components in the school system may have a beneficial effect on public health by increasing overall physical activity among adolescents and possibly among girls and low-active adolescents in particular.

## Background

A decline in physical activity with increasing age has seemed to be a consistent finding in physical activity epidemiology [1,2]. To combat this unfavorable development, the school has been regarded as an advantageous context for health promoting initiatives. Schools may be the only means to reach a large number of young people from diverse socio-economic backgrounds [3]. Although the value of school-based interventions to promote physical activity has been emphasized in several recent reviews, the effects of the strategies and methodology applied have been questioned [4-6]. Furthermore, until recent years physical activity in children and adolescents has primarily been assessed by questionnaires, yielding several weaknesses [7]. Objectively measured physical activity reduces bias and is preferred over subjective methods such as questionnaires. In a recent systematic update of reviews, Kriemler et al. (2011) confirmed the public health potential of high quality, school-based interventions for increasing physical activity in healthy youth, but highlighted that the effect of the reviewed interventions was mostly seen in school-related physical activity while effects outside of school were often not observed or assessed [8]. Cox et al. (2006) stated that physical activity outside of the school environment is a key contributor to a child’s overall level of physical activity and emphasized the need for interventions targeting family and the community as well as the school environment [9]. The most recent reviews have concluded that there is still a lack of high quality school-based interventions on change in physical activity, using objective measures of physical activity among the whole study sample [4,6,8].

Another question that has been raised with regards to recent school-based interventions is for whom interventions are effective. One intervention strategy may not cover the diverse needs of various subgroups, and interventions tailored to specific groups have been suggested and tested with diverging results [6]. It has been a concern when designing interventions that the intervention strategies might not reach the ones that need the efforts the most, e.g. interventions aiming at increasing physical activity might not reach the least active participants but make the active participants even more active. Yildirim et al. (2011) identified gender as the most common moderator of school-based interventions aimed at energy balance related behaviors, and pointed out that girls seem to respond better to such interventions [10]. Previous studies and reviews support this finding, reporting that obesity prevention interventions seem to be more successful among females [11,12]. Nevertheless, in a review of young peoples’ views of effective interventions, Rees et al. (2006) showed that adolescent girls in particular identified barriers to physical activity provided in school. Also, baseline values regarding outcome variables, initial weight status and socioeconomic status have been identified as potential moderators in interventions targeting energy balance related behaviors [10]. Recent reviews have concluded that there is still a lack of knowledge concerning which interventions work for whom, and further investigation of underlying mechanisms of intervention effects have been suggested [6,10,13].

Earlier findings from the HEalth in Adolescents (HEIA) study have shown intervention effects on psychological and social-environmental determinants of physical activity [14] and on sedentary behavior such as watching TV/DVD during weekdays and playing computer games during weekend days after 8 months of intervention [15]. Gender, parental education and weight status moderated these effects. The aim of the present study is to investigate the intervention effects after 20 months of intervention on accelerometer assessed physical activity, and to explore if the intervention reached *a priori* identified subgroups differently; namely girls, participants that are overweight, have parents with low education level or who currently have a low physical activity level.

## Methods

The HEIA study, a school-based multicomponent cluster randomized intervention study (2 academic years), was developed based on the current best practice knowledge to ensure effect on core outcomes (healthy weight development, increased physical activity, reduced sedentary time and a healthier diet), feasibility and sustainability of the intervention program in the public school system [16]. The HEIA study is based on a socio-ecological framework that aims to combine personal, social and physical environmental factors hypothesized to influence overweight and obesity in children, mediated by dietary and physical activity behaviors [17]. The design and procedure of the HEIA study are thoroughly described elsewhere [16]. The CONSORT Statement for reporting a randomized trial is followed according to applicability ( http://www.consort-statement.org).

### Study design and subjects

**Figure 1:**
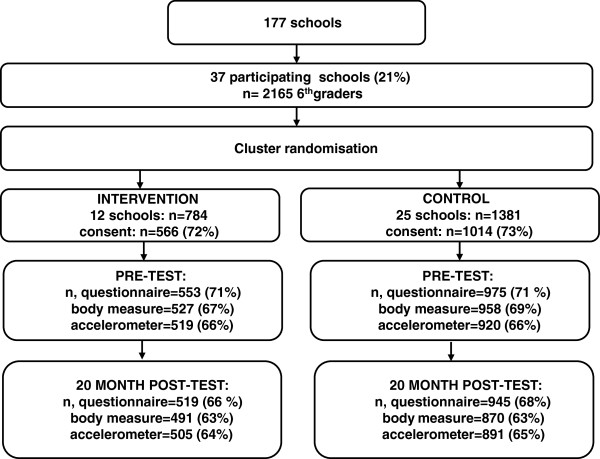
Flow diagram of recruitment, randomization and participation of adolescents in the HEIA study.

Eligible schools were those with more than 40 pupils in 6th grade and located in the 3–4 largest towns/municipalities in 7 counties in south-eastern Norway. Of 177 schools invited, 37 schools agreed to participate. All 6th graders (11–12 year olds) in these 37 schools (n = 2165) were invited to participate. Of these, 1580 (73%) adolescents returned a parent signed informed consent form. Twelve schools were randomly assigned by simple draw to the intervention group (n = 784) and 25 schools to the control group (n = 1381). Figure 1 shows randomization and participation in the HEIA study. Neither participants nor investigators were blinded for condition.

At baseline, 1528 adolescents completed the survey, of which 1439 were present and willing to wear an accelerometer, and of which 1129 (79%) obtained accelerometer data that were regarded as valid according to pre-set criteria in the study. At post intervention, 1418 completed the survey, and 1396 accelerometers were worn resulting in 892 (64%) participants with valid accelerometer data.

The main baseline data collection was conducted by trained staff at each school in September 2007. On the day of the survey, the participating adolescents completed an examination of anthropometric measures, and they filled in an Internet-based questionnaire and a short paper questionnaire about pubertal status. Physical activity was measured objectively by accelerometers. The physical activity data collection was performed separately from the main data collection due to logistics, and the baseline collection of accelerometer data took place from September until the beginning of December 2007. The post intervention main survey took place in May 2009, and the accelerometer assessments were conducted from March to the middle of May 2009.

Ethical approval and research clearance was obtained from the Regional Committees for Medical Research Ethics in Norway and from the Norwegian Social Science Data Service.

### Intervention

Multiple efforts were made and targeted to promote participants’ overall physical activity and to reduce sedentary behavior during the 20 month intervention period (outlined in Table 1 and further described elsewhere [16]). The HEIA study also included intervention strategies to promote a healthy diet, described in Table 1, but these are not further commented on in this paper. Through collaboration with school principals and teachers, and school health services and parent committees, the intervention efforts were orchestrated to increase participants’ physical activity during school hours and in leisure time in order to reduce screen-time activities such as watching TV/DVD, playing computer games, etc. 

**Table 1:** The HEIA-study: Intervention components implemented in 6th and 7th grade in 12 Norwegian schools in 2007–2009

**Setting/arena**	**What**	**Timing**	**Purpose**
**Class (Initiated by classroom-teachers)**	**Lessons with student booklet:**	Once per month - 6th grade winter/spring	Increase awareness of behavior-health relationship, recommended intake levels and own intake
	1. Diet and physical activity		
	2. Meals		
	3. 5 a day		
	4. Sugar rich beverages		
	5. Your choice		
	**Posters for classrooms**	Monthly - throughout the intervention	As a daily reminder of main messages (topic matched fact sheets to parents)
	- Key messages, A4-size, placed on a larger “frame-poster” including the HEIA logo		
	**Fruit and vegetable (FV) break**	Once a week – throughout the intervention	Increase FV intake; cut, serve, taste and eat FV with class mates
	- Cutting equipment per class provided, students brought FV		
	**Physical activity (PA) break**	Once a week – throughout the intervention	Increase PA; introduce PA also outside of PE and by classroom-teachers
	- 10 minutes of PA conducted in regular classrooms, booklet with ideas and CD provided		
	**Sports equipment for recess activities**	Every day - throughout the intervention (some equipment refill at beginning of 7th grade)	Increase PA; stimulate PA during recess – especially among those who do not play ball games
	- 1–2 large boxes per school. Examples of content: Frisbees, jump-ropes, elastic bands, hockey-sticks, a variety of balls		
	**Active commuting campaigns**	5 x 3 weeks: 6th grade: fall, winter and spring	Increase PA; stimulate activity
	- Register days with active transport to/from school for 3 weeks (5 campaigns)	7th grade: fall, winter	
	**Pedometer:**	7th grade	Increase awareness about PA level; stimulate activity
	- One class-set per school to be used in PE (SPARK), as tasks at school, as home assignment and active commuting		
	**Computer tailored individual advice**	7th grade	Increase awareness of;
	1. Fruit	Fall	- recommended intake and PA level
	2. Vegetables	Fall	- own intake of FV, PA level and hours of screen time
	3. Physical activity	Winter/spring	Received personal advise about what and how to change
	4. Screen time	Winter/spring	
	5. Sugar sweetened beverages	Winter/spring	
	+ one-week action plans for each topic (instruction on what, where and when to try one of the pieces of advice for behavior change)		
**Home/parents**	**Fact sheets**	Monthly - throughout the intervention, one behavior per fact sheet	To stimulate parents to evaluate and change the home environment with regards to facilitating or regulating the targeted behaviors
	Facts on targeted behaviors. Practical tasks/challenges for leisure time/weekends in 7th grade		
	**Brochures/information sheets**	Once	To ensure that the fact sheets were read and discussed/applied to the home environment
	Teachers were provided info sheets about the FV break that they could use to inform parents about these		
	**Brochures**:	Once	To provide knowledge and inspiration
	- “Cutting FV”		
	- “Meals – a value worth fighting for”. Handed out together with related fact sheets		
**School wide**	**Kick-off meetings at each school**	Once a year - 6th and 7th grade (fall), 2–3 hours each time	To inform the school management, teachers, school nurse and parent committees about the project and establish/inform the grade level teachers as the “HEIA-team” at school
	- Teacher manuals presented, practical activities tested, material partially provided		
	**Inspirational courses for PE teachers**	Once a year - 6th and 7th grade (fall), 6 hours each time	Teacher-training for PE teachers; methods/activities to increase activity time, enjoyment and self-efficacy for all students during PE classes
	- SPARK ideas/principles [20]		
	**Resource box for school management**	Optional	Focus on healthy food/drinks offered in school/during school events
	**-** Offer to order free tool box for cutting and selling FV		
	**Committee meetings**	Optional	Aimed to stimulate easy-to-do changes on the school grounds that could stimulate activity (booklet/ideas provided). Increase awareness of healthy foods and beverages
	-Meetings with school environment groups/parent committees		
Leisure time activities (NGO’s)*	**Information folder and offer to receive a resource box with equipment for cutting and selling FV**	7th grade (fall)	Create awareness about leisure time activity leaders as role models for dietary habits, to reflect upon availability of food/drinks during practices and special events (i.e. tournaments, weekend training sessions, etc.)

*FV*, fruits and vegetables, *PA*, physical activity, *PE*, physical education, *NGO*, non-governmental organization. *Not successfully implemented.

A kick-off meeting for the teachers was held at each intervention school at the beginning of each school year to inform and encourage the efforts launched, as the teachers were the key persons to implement the intervention efforts. Briefly, the teachers were responsible for holding one structured lecture on energy balance for the students, initiating “HEIA-breaks” - a 10 minute physical activity break during class at least once a week, hanging up “HEIA-posters” in the classrooms, carrying out active commuting campaigns, handing out fact sheets to parents once a month (including student-parent tasks in 7th grade), and implementing a computer tailored program [18] (in 7th grade only) for the students. The intervention schools received an “Activity box” with sports equipment and toys (such as balls, hockey-sticks, jump ropes, Frisbees, etc.) to promote physical activity during recess. Teachers received two inspirational courses in physical education (PE) based on the SPARK program [19] to encourage high intensity and enjoyment for all during PE, one course in 6th grade and one in 7th grade. The intervention strategies were aimed to increase the total physical activity level of all participants in general and to specifically reach the least active participants, in particular inactive girls.

### Outcome measures; physical activity

The children were instructed to wear the accelerometers (ActiGraph models 7164 and GT1M, ActiGraph, Pensacola, FL, USA) all waking hours for five consecutive days except when doing water activities (monitors are not waterproof). The output was sampled every ten seconds for two weekdays and two weekend days. The registration was set to start the second day of wearing the monitors to avoid excessive activity likely to occur during the first day of wearing the device. After collecting the accelerometer, the stored activity counts were downloaded to a computer and analysed by the customized software programs “CSA analyzer” and “Propero” (University of Southern Denmark, Odense, Denmark). In the analyses of accelerometer data only daytime activity (06:00–24:00 hours) was included. Sequences of 20 minutes or more of consecutive zero counts were interpreted to represent non-wear-time and were excluded from each individual’s recording. Data were considered valid if a child had at least three days (including one weekend day) with at least eight hours (480 min) of activity recorded per day. Reasons for not being included in the accelerometer analysis were: not wearing the accelerometer (baseline n = 40, post intervention n = 121), failing to achieve at least three days of assessment (including at least one weekend day) (baseline n = 247, post intervention n = 378) and instrument malfunction (baseline n = 23, post intervention n = 5). The adolescents with valid accelerometer data at both baseline and post intervention (n = 700) are included in this paper. A secondary analysis was done including those registering only for two days, in order to investigate the impact of this attrition.

Sedentary time was defined as activity at intensities less than 100 counts per minute (cpm), and expressed as min/day of accelerometer activity measured which equals the intensity of sitting or lying down (<1.5 MET) [20]. Activity recordings at intensities between 100–2000 cpm were defined as light activity, reflecting activities as standing, walking slowly or easy play. Moderate to vigorous activity (MVPA) was defined as all activity at intensities above 2000 cpm. This threshold is approximately equivalent to a walking pace of 4 km/h in youth [21]. These cut off points have been used in previous studies [22,23]. Sedentary time, light activity and MVPA were expressed as min/day of accelerometer activity measured.

The average number of minutes that the participants wore the accelerometer and the number of activity counts per minute (cpm) were calculated, and mean cpm (mcpm) was used as the main outcome variable. Mcpm as a summary measure of total physical activity in children is commonly used and has been validated against the “gold standard measurement” doubly labelled water and found valid [24]. Since outcomes on mcpm measured by model 7164 and GT1M have shown to differ [25], a free-living validation study of the monitors used in the HEIA study was conducted (Grydeland et al., unpublished observations). As model 7164 showed to measure 11% higher total mcpm than GT1M, a correction factor of 0.9 was applied to the total mcpm from model 7164 to be comparable to the GT1M outcome. This correction factor was applied to all analyses where mcpm was the outcome. To correct for differences in accelerometer model output in minutes spent at different intensity level, a dummy variable was entered into the analyses to adjust for accelerometer model/combination.

Estimate categories were made to detect potential differences in “at school activity” (08:00–15:00) and “after school activity” (15:00–22:00). These estimates were based on accelerometer recordings on weekdays only. The participating schools started and ended school hours at different hours, but no school started before 08.15 hours or ended later than 15.00. Only one school ended at 15.00 hours on one weekday, all else ended earlier. Commuting time is therefore included in “at school activity” time. Participants with mcpm below the median value (mcpm = 480) at baseline were categorized as “low-activity group” and participants above median as “high-activity group”.

### Anthropometric and demographic measures

Height and weight were measured by trained staff according to standard procedures. Body mass index (BMI) was calculated as weight/(height × height) (kg/m^2^). The age- and gender specific BMI cut-off values proposed by the International Obesity Task Force [26] were used to categorize the adolescents as normal weight or overweight. As only 1.9% of the participants at baseline were obese these were included with the overweight in the analyses. The pubertal scale utilized in the study is based on the Pubertal Category Scores (PCS) [27].

Parents reported their educational level as part of the informed consent for their adolescents. Parental education was categorized into three levels: high-school (12 years or less), university/college <3 years (between 13 and 16 years), and university/college >3 years (16 years or more). The information about education from the parent with the highest education was used in the analyses, or else the one available.

### Power calculations

The power calculations were primarily based on the main outcome of the HEIA study; changes in BMI, and secondary changes in the addressed behaviors; intake of fruit, vegetables and soft drinks and physical activity [16]. Taking the cluster effect of randomly assigning schools to intervention and control into account, assuming that 80% of the pupils would take part, an attrition rate of maximum 15% per year, we aimed for 40 schools with an average of 45 pupils participating from each school (n = 1800). The final sample was lower (n = 1580), but the attrition rate per year was only 4%. We concluded that the final sample should have power enough to detect a difference between intervention and control schools after two years. For accelerometer assessed physical activity, a difference of 62 cpm was used in the power analyses, based on a nationally representative population study on 9- and 15-year olds [23].

### Data preparation and statistics

For descriptive statistics and dropout analysis, independent t-tests and chi-square tests were used to examine differences between groups (Table 2). The effect analyses were conducted in linear mixed models to be able to take the clustering effect of sampling by school into account. The effect was estimated by a regression of post-test values of mcpm (or other outcome variables) on condition, adjusted for grand mean centered baseline values of mcpm (or other outcome variables). In the main effect analyses (Table 3) a few extreme outliers were replaced by the mean value + 3SD as suggested by Field [28]. All effect analyses were adjusted for covariates and confounders; gender, pubertal status, weight status, month of measuring physical activity and parental education. Analyses were also performed to detect differences in activity on weekdays and weekend days. Intervention effects on time spent at different intensity levels were also tested. Subgroup analyses were performed on gender, weight category, activity category and by parental education category to explore potential differences in effect of the intervention by these subgroups. These subgroups were pre-specified based on the nature of the study (trying to affect the least active and girls in particular). We expected girls to be more conscientious to the intervention components than boys [10,11], the least active participants to have a larger potential for change, and the overweight and participants of parents from the lowest parental education category to be harder to affect [10]. The significance level was set to 0.05. Data were analysed using the IBM SPSS, version 18 (SPSS Inc., Chicago, IL, USA). 

**Table 2:** Baseline characteristics for the HEIA-study participants [Mean (SD) or %]

	**Intervention group (n = 215)**	**Control group (n = 485)**	**p**
**Age** (years)	11.2 (0.3)	11.2 (0.3)	0.3
**Girls** (%)	54	60	0.2
**BMI** (kg/m^2^)	18.0 (2.7)	17.9 (2.7)	0.7
**Overweight/obesity**^**a**^ (%)	13	14	0.7
**Puberty scale score** (%)			
Pre-pubertal	17	19	0.8
Early pubertal	34	35	
Mid-late-post pub.	49	47	
**Parental education** (%)			
<12 years	25	33	0.08
13-16 years	34	34	
>16 years	40	33	

^a^As defined by International Obesity Task Force’s cutoffs for overweight/obesity at age from 10.5 to 12.5 [26].

**Table 3:** Physical activity in the HEIA intervention- (n = 215) and control group (n = 485), and intervention effect*

	** BASELINE**		** POST-INTERVENTION**		INTERVENTION EFFECT*	
	**Control**	**Intervention**	**Control**	**Intervention**		
**Counts/min**	**Mean (SD)**	**Mean (SD)**	**Mean (SD)**	**Mean (SD)**	**Estimate (CI)**	**p**
**Overall PA, all (n = 700)**	511 (146)	473 (146)**	564 (255)	570 (252)	50 (−0.4, 100)	**0.05**
PA weekdays	553 (165)	509 (164)**	573 (233)	574 (255)	35 (–14, 83)	0.16
PA weekend days	453 (197)	424 (180)	549 (356)	560 (353)	60 (−15, 136)	0.11
Estimated PA at school	621 (189)	604 (188)	582 (223)	559 (208)	2 (−56, 60)	0.94
Estimated PA after-school	504 (248)	432 (217)**	599 (381)	622 (421)	69 (−20, 144)	0.13
**Overall PA, girls (n = 392)**	478 (128)	464 (151)	506 (230)	535 (234)	65 (5, 124)	**0.03**
PA weekdays	514 (140)	496 (171)	517 (207)	551 (252)	54 (−3, 111)	0.06
PA weekend days	431 (193)	418 (185)	488 (316)	505 (292)	74 (−12, 159)	0.09
Estimated PA at school	561 (170)	559 (186)	500 (182)	527 (181)	30 (−32, 92)	0.34
Estimated PA after-school	480 (213)	453 (239)	565 (352)	608 (416)	81 (−18, 181)	0.11
**Overall PA, boys (n = 308)**	549 (157)	488 (137)**	632 (268)	622 (268)	32 (−35, 99)	0.35
PA weekdays	598 (181)	528 (152)**	639 (244)	608 (257)	12 (−52, 76)	0.72
PA weekend days	478 (200)	434 (173)	622 (388)	643 (417)	32 (−75, 139)	0.55
Estimated PA at school	691 (186)	673 (170)	679 (228)	606 (237)	−40 (−119, 40)	0.32
Estimated PA after-school	532 (281)	401 (177)**	639 (410)	643 (429)	37 (−70, 144)	0.50

*PA*, physical activity. * Effect analyses were adjusted for school clustering, baseline physical activity, gender, pubertal status, month of measuring physical activity, weight category and parental education. ** Intervention group means significantly lower than control group means, p < 0.01. Test of interaction condition x gender: p = 0.22.

## Results

Dropout analyses showed no differences with regard to age, BMI, weight category or parental education between the participants who provided valid accelerometer measures at both time points (n = 700) against the ones who did not provide valid accelerometer measures at both time points (n = 828). There were, however, significantly more boys in the group without valid accelerometer measures (p < 0.001).

There were no significant differences between the intervention and control group at baseline for anthropometric or socio-demographic values (presented in Table 2).

Table 3 shows physical activity at baseline and post intervention and intervention effects. The intervention had an effect on total physical activity at the level of p = 0.05, with a net effect between intervention and control of 50 cpm in favour of the intervention group (95% Confidence Interval −0.4, 100. Mean (SD) accelerometer wear time at baseline was 780 (61) min/day and 793 (58) min/day for intervention and control groups, respectively, with corresponding numbers for post intervention of 771 (73) min/day and 792 (66) min/day. We did rerun the analysis on total physical activity including n = 178/n = 235 subjects having registered accelerometer data for only two days at baseline and post intervention, respectively. The results from this analysis were of the same magnitude as when applying the full sample (three days registration) of this study (Effect estimate 52 (CI −0.03, 104), p = 0.05). The subgroup analyses indicated a significant effect in girls (p < 0.03) but not in boys (p = 0.35).

Change in physical activity pertaining to intensity levels is shown in Table 4. There was no significant intervention effect for time spent sedentary between the intervention group and the control group (p = 0.16). At baseline both intervention and control participants spent on average 63% of the monitored time sedentary, and both groups had an increase in time spent sedentary from age eleven to 13. Stratified gender analyses revealed a significant intervention effect for girls of 22 minutes (CI 5, 124, p = 0.03) for time spent sedentary, reflecting a significantly smaller increase in sedentary time among girls in the intervention group versus the control group. No similar effect was seen among boys.

**Table 4:** Minutes distributed at intensity levels in the HEIA intervention- and control group, and intervention effect*

	** BASELINE**		** POST-INTERVENTION**		INTERVENTION EFFECT*	
	**Control**	**Intervention**	**Control**	**Intervention**		
**Minutes in:**	**Mean (SE)**	**Mean (SE)**	**Mean (SE)**	**Mean (SE)**	**Estimate (CI)**	**p**
**All (n = 700):**						
Sedentary activity	495 (3.0)	496 (4.6)	519 (3.4)	506 (5.2)**	−14 (−33, 6)	0.16
Light activity	229 (1.8)	224 (2.9)	202 (2.0)	195 (3.1)**	−5 (−15, 5)	0.33
MVPA	68 (1.0)	63 (1.6)**	71 (1.3)	67 (2.0)	2 (−3, 7)	0.45
**Girls (n = 392):**						
Sedentary activity	499 (4.0)	496 (6.0)	533 (4.3)	510 (6.1)	−22 (−43, -2)	**0.03**
Light activity	229 (2.3)	221 (3.5)	201 (2.6)	193 (3.8)	−3 (−14, 9)	0.63
MVPA	62 (1.2)	60 (1.8)	62 (1.4)	62 (2.0)	5 (−2, 12)	0.13
**Boys (n = 308):**						
Sedentary activity	490 (4.3)	495 (7.3)	502 (5.4)	499 (8.7)	−9 (−36, 18)	0.50
Light activity	228 (2.9)	228 (4.9)	202 (3.2)	197 (5.1)	7 (−20, 7)	0.33
MVPA	75 (1.7)	68 (2.9)	81 (2.2)	75 (3.5)	1 (−10, 7)	0.77

Intervention group n = 215, control group n = 485. Sedentary activity <100 cpm, Light activity ≥100 < 2000 cpm, MVPA ≥2000 cpm. MVPA: moderate to vigorous physical activity. Mean values are adjusted for accelerometer model at baseline and post intervention. * Effect analyses were adjusted for school clustering, baseline physical activity, gender, pubertal status, accelerometer model, month of measuring physical activity, weight category and parental education. ** Intervention group means significantly lower than control group means, p < 0.05. Test of interaction condition x gender: p = 0.22.

Table 5 shows mcpm and intervention effect with participants grouped by baseline activity level and weight status. In the low activity group there was a significant overall positive intervention effect of net 92 cpm (CI 41, 142, p < 0.001), while no effect was seen in the high activity group. The intervention participants in the low-activity group showed a significant net increase of 96 cpm compared to the control group during weekdays (Effect estimate 96 (CI 46, 145) p < 0.001), whereas no intervention effects were seen during weekend days (data not shown). There was no intervention effect during school hours. Regarding after school hours physical activity, participants in the low-activity category from the intervention group had a net increase of 159 cpm more than the control group (Effect estimate 159 (CI 77, 241) p < 0.001). There was no intervention effect on participants in the high-activity category (data not shown).

**Table 5:** Physical activity by baseline activity level and weight status, and intervention effect*

	** BASELINE**		** POST-INTERVENTION**		INTERVENTION EFFECT*	
	**Control**	**Intervention**	**Control**	**Intervention**		
**Counts/min: All (n = 700)**	**Mean (SD)**	**Mean (SD)**	**Mean (SD)**	**Mean (SD)**	**Estimate (CI)**	**p**
Low-activity group (n = 350)	392 (66)	373 (59)**	499 (231)	557 (261**)§**	92 (41, 142)	**<0.001**
High-activity group (n = 350)	615 (114)	608 (115)	621 (263)	587 (239)	10 (−67, 87)	0.79
Normal weight (n = 591)	517 (142)	482 (146)**	565 (252)	585 (248)	62 (10, 115)	**0.02**
Overweight (n = 93)	468 (160)	406 (115)	566 (283)	432 (173)	−96 (−211, 19)	0.10

* Analyses were adjusted for school clustering, baseline physical activity, gender, pubertal status, month of measuring physical activity, weight category and parental education. ** Intervention group mean significantly lower than control group mean, p < 0.05. **§** Intervention group mean significantly higher than control group mean, p < 0.05. Test of interaction condition x activity level: p = 0.16, condition x weight status: p = 0.16.

Categorized by weight status, the analyses show that the normal weight in the intervention group increased their physical activity significantly more than the normal weight in the control group, with a net increase of 62 cpm (CI 10, 115, p = 0.02). Physical activity during weekdays and weekend days, and during school hours and after school hours was investigated, but no differences were found between groups (data not shown).

Finally, effect analyses were also run for participants stratified by level of parental education (Table 6). There were no intervention effects for participants with parents having less than twelve years of education and for participants with parents having more than 16 years of education. But, for participants with parents in the middle parental education level category of 13–16 years of education, we found a significant intervention effect on overall physical activity level (Effect estimate 98 (CI 17, 178) p = 0.02) and for physical activity during weekend days (Effect estimate 157 (CI 43, 271) p = 0.008) in favour of the intervention group.

**Table 6:** Physical activity by level of parental education and intervention effect*

	** BASELINE**		** POST-INTERVENTION**		INTERVENTION EFFECT*	
	**Control**	**Intervention**	**Control**	**Intervention**		
**Parental education/Counts/min**	**Mean (SD)**	**Mean (SD)**	**Mean (SD)**	**Mean (SD)**	**Estimate (CI)**	**p**
**≤12 years (n = 211)**	504 (156)	481 (141)	559 (278)	554 (236)	43 (−37, 123)	0.29
PA weekdays	564 (177)	538 (158)	563 (240)	570 (249)	38 (−40, 115)	0.34
PA weekend days	421 (196)	405 (167)	551 (370)	538 (364)	55 (−53, 163)	0.31
Estimated PA at school	637 (202)	645 (197)	602 (246)	557 (185)	−6 (−77, 66)	0.88
Estimated PA after-school	508 (245)	457 (208)	550 (353)	625 (403)	107 (−9, 223)	0.07
**13-16 years (n = 236)**	505 (141)	465 (145)**	568 (233)	617 (284)	98 (17, 178)	**0.02**
PA weekdays	537 (149)	500 (161)	595 (225)	597 (272)	39 (−32, 109)	0.27
PA weekend days	454 (198)	415 (176)	529 (331)	621 (381)	157 (43, 271)	**0.008**
Estimated PA at school	593 (178)	588 (199)	591 (197)	576 (221)	27 (−40, 94)	0.41
Estimated PA after-school	500 (257)	424 (193)**	646 (419)	665 (485)	47 (−98, 192)	0.52
**>16 (n = 240)**	521 (136)	478 (152)**	556 (252)	546 (230)	2 (−90, 94)	0.96
PA weekdays	552 (159)	501 (170)**	551 (228)	561 (248)	31 (−43, 104)	0.40
PA weekend days	483 (193)	444 (194)	562 (366)	529 (321)	−28 (−166, 109)	0.67
Estimated PA at school	632 (182)	596 (171)	551 (226)	543 (214)	−13 (−92, 66)	0.74
Estimated PA after-school	496 (226)	426 (246)**	580 (347)	593 (376)	50 (−53, 153)	0.33

* Effect analyses were adjusted for school clustering, baseline physical activity, gender, pubertal status, month of measuring physical activity and weight category ** Intervention group means significantly lower than control group means, p < 0.05. Test of interaction condition x parental education: p = 0.03.

## Discussion

The present study showed an intervention effect on overall physical activity at the 5% alpha level. The intervention effect appeared to be more profound among girls, and among participants in the low-activity group compared to boys and to participants in the high-activity group, respectively. Further, the intervention appeared to have a stronger effect among normal weight participants and participants with parents reporting 13–16 years of education compared to their counterparts.

With an intervention effect at alpha level 0.05 there is a degree of uncertainty to the results that needs to be considered. There is a 5% chance that the findings are not attributed to the intervention, which means the greatest value of uncertainty conventionally accepted before the findings are dismissed as non-significant. Keeping this in mind, the intervention effect on total physical activity is somewhat in contrast to results from the KISS intervention; a Swiss cluster randomized controlled school based physical activity programme. The KISS study, while comprising a bit younger participants, showed a favourable intervention effect on moderate to vigorous activity at school and all day, and also on total physical activity at school, but no effect on overall daily physical activity [29]. No intervention effect on overall physical activity was shown in the Danish CoSCIS study either, with an intervention including a doubling of time for PE among 6–7 year olds [30]. Compared to the KISS programme and the CoSCIS study, the HEIA intervention had less promotion of high intensity activities but focussed on increasing overall physical activity. While the HEIA study used a multi-facetted approach to increase physical activity including several small reminders and opportunities to increase all day physical activity level, the KISS study was oriented toward PE and using expert PE teachers and extracurricular mandatory PE. The CoSCIS study also used PE as their main intervention component, including a doubling of lessons per week, teacher training and an upgrade of PE and playing facilities. From the effect analyses it is not possible to disentangle specific intervention components to account for our findings. Some intervention components may have been more effective than others, or results may reflect synergistic effects of the intervention program as a whole. Thus, in concordance with suggestions in recent reviews [6,8], the HEIA study aimed to affect physical activity in adolescents through multiple components and by combining personal, social and physical environmental factors. The increase in physical activity from baseline to post intervention in the control group was unexpected, as previous literature has shown decreasing physical activity with increasing age in youth [1,23,30]. Since both groups increased, an increase as a result of the intervention may have been harder to detect, yet the intervention showed a positive effect. The intervention group was significantly less active than the control group at baseline, and it can be argued that the intervention group had a larger potential for change. However, these issues were taken care of by controlling for baseline-values in the effect analyses.

The relatively large increase in physical activity from baseline to post intervention in both groups can be attributed to seasonal variation. The baseline physical activity assessment was conducted during fall and post intervention assessment during spring. Kolle et al. (2009) observed seasonal variations in physical activity among 9 year old Norwegian children, but not among 15 year olds [31]. The intervention effect should, however, not be affected by season, as both groups were measured simultaneously. The increase might also be a result of contamination effects of being the control group in a study aimed at increasing physical activity. When recruiting schools, most schools stated that they were hoping to become an intervention school to receive the intervention efforts. This could have stimulated the control schools to initiate their own “intervention”.

The overall increase in physical activity from baseline to post intervention was seen both on weekdays and weekend days, but with a larger increase on weekend days. The larger increase during weekend days may reflect the larger potential for change since the baseline values within that period of the week were considerably lower than during weekdays. The intervention components addressed both weekday and weekend day activity. The finding that the physical activity level was higher during weekdays than weekend days is consistent with earlier cross-sectional findings from Norwegian 9 and 15 year olds [23].

The participants’ mean distribution of activity in our study differed between the two time points. Physical activity during school hours declined and physical activity after school hours and during weekend days increased for both groups and both genders. The decline in physical activity at school might be due to more demanding school curricula in 7th grade than 6th grade, and happened despite several intervention efforts aimed at increasing physical activity at school. A reason for the demonstrated decline in physical activity during school hours may also be a lack of facilities perceived as attractive by the adolescents as they grow older. Nettlefold et al. (2011) studied physical activity during the school day in Canadian 8–11 year olds and observed low physical activity during parts of the school day [32]. The authors pointed out an urgent need to increase the intensity of activity during PE, and to provide more and/or facilitated opportunities for physical activity during school breaks. Haug et al. (2010) found that outdoor facilities in Norwegian secondary schools were associated with students’ daily physical activity participation during school breaks [33]. Students in schools with many facilities had significantly higher odds of being physically active compared to students in schools with fewer facilities [33]. The activity increase in both groups after school hours is hard to explain. A possible reason may be increased volume of exercise in leisure time sports activities with increasing age. The participants may also have been stimulated to increase leisure time physical activity in line with the HEIA study aims. There was, however, no intervention effect on these outcomes. Concerning time spent at different intensity levels, no intervention effect was seen for time spent in MVPA. Nevertheless, this was not a targeted aim of the study. However, reducing sedentary time was a clear aim of the study but no intervention effect was seen for the total sample. Exploring subgroups, boys appeared to have higher overall physical activity on all time points than girls, but the difference in increase from baseline to post intervention was significantly higher among girls in the intervention group compared to girls in the control group. The gender difference in intervention effect was also seen with time spent at different intensity levels as outcome. Girls in the intervention group increased significantly less in sedentary time from baseline to post intervention than girls in the control group. This is promising, as a recent comprehensive systematic review revealed a dose–response relationship between increased sedentary behaviour and unfavourable health outcomes in school-aged children [34]. When the intervention strategies were planned and developed, the study group had a particular focus on making sure that it should appeal to inactive girls. By offering low threshold activities the aim was to make the physically less active participants want to take part rather than fear to take part. Intervention strategies aimed to target certain groups have earlier showed diverging results [6]. These results suggest that having an inclusive approach but focusing on certain subgroups within the intervention can be successful. However, when interpreting the findings one should be aware of the lack of significant interaction between condition and gender. When an interaction term shows p < 0.1 subgroup analysis is conventionally required for statistical reasons. We based our subgroup analyses on pre-specified hypotheses based on the nature of the study and previous findings [10,14,15]. To evaluate the credibility of subgroup analyses Sun et al. (2010) have suggested eleven criteria [35]. By meeting most, but not all these criteria, we find support for doing these secondary investigations, but we also acknowledge a degree of uncertainty of these exploratory findings.

Gender aside, the intervention appeared to affect other subgroups differently as well. The intervention participants in the low-activity group demonstrated a significant increase in physical activity from baseline to post intervention. These results are encouraging, as increasing the activity level among the least active can cause larger health benefits than among participants already active [36]. As a decline in physical activity with increasing age can be expected [1,23], it is also noteworthy that we did not observe a significant decrease in the high-activity group. Among those overweight, the participants in the control group were more active at both time points and had a more positive development than participants in the intervention group. The issue of different responses on different groups are discussed by Brown and Summerbell (2009) in a comprehensive review on obesity-prevention in school-children [11]. They suggest that particularly boys and girls and those differentiating in weight status in the age range of 10 to 14 seem to respond differently to different elements of the interventions [11]. Participants from different parental education categories were also affected differently by the intervention. An intervention effect was observed only among participants with parents having a “mid-range” educational level. However, investigating other outcomes in the HEIA study, Bjelland et al. (2011) found no moderating effects of parental education for boys or girls with respect to intake of sugar-sweetened beverages, time used for watching TV/DVD and computer/game-use [16]. The results of this intervention study are important to public health, as feasibility and sustainability were high priorities when designing the intervention. This has been recommended in previous studies and reviews [6,8,37]. Although comprehensive, the intervention components were designed to be able to fit into current school curricula without substantial extra costs. With limited instructions and material provided by the study group, teachers were key deliverers of the intervention components. No extra personnel or costly material are needed to carry out such components in the current school system, and all components could easily be incorporated into existing curricula for this age group.

### Strengths and limitations

The strengths of the present study include the study design and the large number of participants. The multicomponent intervention, lasting 20 months, was designed to be feasible to the school system and not financially demanding. Also, measures including objectively assessed anthropometric measures, pubertal maturation, self-reported parental education and whole sample measurement of physical activity by accelerometers are clear strengths of this study.

We acknowledge that our study has several limitations. Firstly, the use of two different generations of accelerometers (for practical reasons) represents an element of uncertainty compared to using only one kind. To address this issue we explored the potential difference between generations of monitors, and adjusted the values accordingly. Secondly, at baseline physical activity was assessed during fall and at post intervention physical activity was assessed during spring. However, the measurement month was adjusted for in the effect analyses, and this issue was also taken care of by the study design. Thirdly, according to the power-calculations of the study [16], the number of participants providing valid accelerometer data at both time points was lower than opted for, and a higher number of participants with valid recordings may have made it easier to detect significant intervention effects on physical activity. However, the power-calculations on physical activity may also have been overestimated, since investigating change in such large groups objectively has rarely been done in previous studies. The large drop-out reduces the generalizability of the results. However, few differences were seen between those who provided accelerometer data at both time points and those who did not. Fourthly, the use of subgroup analysis is criticized by some and called for by others [38]. We chose to include subgroup analyses based on the nature of the study where specific groups were targeted when planning the intervention efforts. Furthermore, the HEIA intervention components were primarily delivered through the teachers at the intervention schools. Unpublished process evaluation revealed that the degree of implementation differed between schools [39], with a reduced dose of intervention received by the participants observed from mid-way to post intervention [14]. Also, when investigating intervention effects of a multi-component intervention, it is not possible to sort out whether or how the components worked separately. Finally, the potential for generalization of our findings might be limited as the sample was recruited from a limited geographic area. However, comparing the HEIA study sample to nationally representative figures for 9 and 15-year-olds, the measures from the participants in the HEIA study lie adequately between the measures of the 9 and 15-year-olds when it comes to objectively measured height, weight and total physical activity [40].

## Conclusions

A comprehensive but feasible, multi-component school-based intervention can affect physical activity patterns in adolescents by increasing overall physical activity. This intervention effect seemed to be more profound in girls than boys, low-active adolescents compared to high-active adolescents, participants with normal weight compared to overweight, and for participants with parents having middle education level as opposed to high and low education level, respectively. An implementation of these intervention components in the school system may have a beneficial effect on public health by increasing overall physical activity among adolescents and possibly among girls and low-active adolescents in particular.

## Abbreviations

BMI: Body mass index; PA: Physical activity; CI: Confidence interval; Cpm: Counts per minute.

## Competing interests

The authors declare that they have no competing interests.

## Authors’ contributions

All authors are responsible for the reported research. MG worked on the statistical analyses, wrote the first draft of the manuscript and made the greatest contribution to the paper. NL was the project coordinator and participated in all parts of the work. KIK, LFA, YO and SAA were mainly involved in designing the study while IHB, MB and MG were mainly responsible for planning and conducting the data collections and the intervention. KIK initiated the study. All authors provided critical revision of the paper, and read and approved the final manuscript.
